# Lymphocytes as a Biomarker of Frailty Syndrome: A Scoping Review

**DOI:** 10.3390/diseases9030053

**Published:** 2021-07-13

**Authors:** Rut Navarro-Martínez, Omar Cauli

**Affiliations:** 1 Haematology Department, Hospital General Universitario, 46014 Valencia, Spain; Rut.Navarro@uv.es; 2 Department of Nursing, University of Valencia, 46010 Valencia, Spain; 3 Frailty and Cognitive Impairment Group (FROG), University of Valencia, 46010 Valencia, Spain

**Keywords:** geriatric evaluation, biomarkers, gender differences, CD4 cells, CD8-cells, immunity

## Abstract

Frailty is a geriatric syndrome characterized by a decrease in physiological reserve and reduced resistance to stress, as a result of an accumulation of multiple deficits in physiological systems. Frailty increases the vulnerability to adverse events and is associated with the aging process. Several studies show an association between frailty syndrome and altered blood lymphocyte levels, which is therefore potentially useful for monitoring interventions to improve or delay frailty. The main objective of this review is to provide an analysis of the current evidence related to changes in lymphocyte counts and their associations with frailty syndrome. To that end, the literature published in this field until March 2021 was in several databases: PubMed, SCOPUS, and Cochrane. Eighteen studies analyzed the association between lymphocyte counts, lymphocyte subtypes, and frailty syndrome. Eighteen studies were analyzed, and most of them reported associations. Interestingly, the association between frailty syndrome and lower lymphocytes counts appears in different clinical conditions. Further studies are needed to determine the sensitivity of lymphocyte counts and lymphocyte subtypes in the diagnosis and monitoring of frailty syndrome, and for this measure to be used as a biomarker of frailty status.

## 1. Introduction

Frailty is a clinical syndrome characterized by a decline in the multisystem functional reserve, which causes greater vulnerability to stressful situations and predisposes one to numerous adverse health effects including falls, hospitalization, disability, and mortality [1]. The prevalence of frailty increases with age [2], and an increasing number of frail older adults is expected due to the progressive aging of the population. Although frailty syndrome is classically associated with aging processes and most studies investigate it in older adults (age 65 years and older), different chronic conditions may promote frailty at earlier ages. For this reason, new interesting studies have also evaluated frailty in younger patients with cancer, diabetes, or coronary heart diseases [3,4,5]. Although numerous ways of measuring frailty syndrome have been described, the most widely accepted operational definition is one that considers frailty as a clinical syndrome that includes the presence of three or more of the following clinical criteria: Involuntary weight loss in the last year, muscle weakness, slow gait, self-reported fatigue, and low physical activity [5]

However, this or other instruments used to assess frailty have limited clinical utility, are time-consuming, are sometimes difficult to perform, and are sometimes not validated or sufficiently standardized [1]. In addition, they identify frailty late, once the clinical manifestations have started [5]. Existing evidence suggests that the physiological deterioration associated with frailty begins to be evident in a preclinical phase [3]. Its early detection would therefore help implement prevention and early intervention therapies in order to treat this syndrome and prevent related adverse results since it has been suggested that frailty could be reversible [6]. Accordingly, it is necessary to develop new tools that not only allow frailty to be identified in its early stages, including before symptoms occur but also the predisposition to develop this syndrome [7].

The search for frailty biomarkers that provide additional information to that obtained from clinical data has therefore become especially important in recent years [8]. Several studies have suggested that the inclusion of laboratory data in frailty indexes could improve its prognostic power [9]. Ascertaining the pathophysiology of the disease is key to the development of frailty biomarkers. The pathophysiological mechanisms underlying the onset and development of frailty remain complicated and poorly understood, but chronic systemic inflammation has been considered one of the most important components contributing to its development [10,11]. In fact, several inflammatory mediators have been consistently associated with frailty [12]. Furthermore, immuno-senescence is believed to be involved in the development of the chronic inflammatory state related to frailty syndrome [12,13]. The main feature of immunosenescence is the change in the cellular composition of the T-cell compartment, which includes a decline in the number of naive phenotype cells and, conversely, an increase in the number of memory phenotype cells, all of which culminates in a proinflammatory state with greater production of cytokines, which leads to lower levels of cell proliferation and greater resistance to apoptosis [14]. One of the characteristic changes of the immune system with age is the alteration of the number and composition of different types of lymphocytes in the circulation. In older people, the number of CD4+ and CD8+ T lymphocytes and B lymphocytes is reduced, whereas the number of NK lymphocytes is increased compared to younger people. At the subset level, there is also a decrease in naïve T and B cells and an increase in memory T and B cells with aging. These changes may reflect a combination of reduced naïve lymphocyte production and accumulation of memory lymphocytes as a result of reduced overall lymphocyte production and host-environment interaction over time. As a result of these changes, older people are more vulnerable to infectious diseases and adverse outcomes when lymphocyte counts are lower [15]. Based on this, recent studies have associated frailty with alterations in the total lymphocyte count and lymphocyte subpopulations [3,16,17,18].

Lymphocyte counts and the different lymphocyte subpopulations may therefore be suitable biomarkers that provide useful information for the early identification of frailty. Its wide availability and low cost also reinforce its potential for use in daily clinical practice, by allowing identification or at least contributing to the early identification of patients at risk of frailty in a simple way, while requesting a routine blood test. The understanding of the immune mechanisms underlying frailty is also of great interest for developing, adapting, or evaluating the modification of prophylactic and/or therapeutic treatments. In this study, a review of the scientific evidence was therefore carried out to determine the relationship between total lymphocyte counts and frailty assessment tools, the associations with specific frailty criteria [5], and whether lymphocytes subpopulations are involved in these associations.

## 2. Materials and Methods

### 2.1. Literature Search

A review of the scientific literature was performed by searching in the electronic bibliographic databases PubMed, Scopus, and Cochrane for research articles published on this topic up to March 2021. The terms used for literature searches were “frailty” AND (Boolean Operator) “lymphocytes”. Some limits were established, e.g., “species: human” for PubMed searches. In the case of Scopus, we excluded studies performed on experimental animals. Cochrane databases reported only results performed in human studies. The database searches were loaded into a bibliographic manager (Refworks), and duplicate articles were identified and removed. The reference lists of all relevant articles were manually reviewed to identify additional articles that were potentially useful for the aims of this study.

### 2.2. Eligibility Criteria

We applied the following inclusion criteria: (1) Recognized as an original article, (2) full text published in English or Spanish, (3) study participants were identified as “non-frail” or “frail” or “pre-frail” in the title, abstract, and/or text, and (4) the study investigated the total count or the different lymphocyte subtypes in relation to frailty syndrome in individuals. We used no limitations on the publication date in the search process. We analyzed the studies analyzing the associations between frailty syndrome and lymphocytes’ count in blood in different clinical situations (from community-dwelling individuals with different comorbidities to patients with specific diseases e.g., cancer, diabetes, or coronary diseases). Manuscripts different from original articles (narrative reviews, comments, and editorials) were excluded.

### 2.3. Data Collection and Analysis

After reading the titles and abstracts of the articles extracted from the databases, we retrieved the full electronic text of those articles that met the eligibility criteria. The following data were extracted for all studies complying with inclusion criteria: First author/year; study design; sample size (*n*); subjects (sex and age); disease/patients; and tool to measure frailty syndrome. In addition, the relationship between lymphocyte count and its subtypes with frailty criteria was carefully analyzed. For each of the retrieved articles, the two authors independently extracted data. Any disagreement between the two reviewers on the documents, or the data extracted from them, was resolved by analyzing again the data simultaneously.

## 3. Results

**Figure 1:**
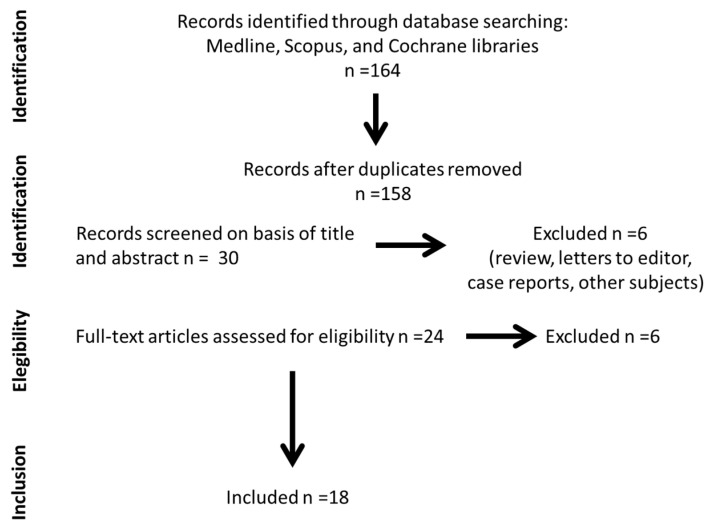
Summary of articles retrieval in scientific databases.

The results of literature searches are shown in Figure 1.

### 3.1. Relationship between Total Lymphocytes and Frailty Syndrome Prevalence

Several observational studies have analyzed the relationship between lymphocytes counts in blood and frailty syndrome as these cells represent an easy and cheap marker related to the chronic low-grade inflammatory state associated with frailty syndrome (Table 1). These studies evaluated frailty syndrome with Fried’s criteria (14 studies), the Edmonton scale (one study), the Rockwood frailty index (one study), the Carolina frailty index (one study), the Frailty index containing 36 “health deficits” (one study), and the modified 11-item frailty index score (one study). With the exception of four studies, where no association was observed, they found that a lower total count or percentage of lymphocytes, even within the normal physiological range, is associated with higher rates of frailty and greater severity. However, the exclusion of men [19,20,21] and the relatively small sample sizes together with the lack of adjustment for possible confounding factors in the statistical analysis [22,23] could partially explain the discrepant results in these studies. The sex differences appear to be important when comparing studies performed only in men or in women, since the prevalence of frailty, although increasing with age in both males and females, is higher in females than in males. The presence of frailty had a negative impact on survival in both men and women, whereas mortality rates are higher in men than in women. Likewise, a multivariate analysis single-center observational study in patients with coronary artery disease showed that relative lymphocyte counts were inversely related to a higher risk of being frail, with an exponential increase in risk [18].

In both geriatrics research and clinical settings, the most-used definitions of frailty were developed by Fried and co-workers (physical frailty phenotype) and Rockwood and co-workers (the frailty index based on accumulative deficits). Among the studies analyzed in the review, the tools mostly used were the Fried model [10,12,13,16,17,18,20,23,24,25,26,27,28], which defines a phenotype that includes the presence of three or more of the following clinical criteria: Involuntary weight loss in the last year, muscle weakness, gait slow, self-reported fatigue, and low physical activity [5]. For their part, Gilmore et al. [29] assessed frailty using a modified version of Fried’s frailty score, using four of the five available criteria (weakness, exhaustion, walking speed, and physical activity). Rockwood’s frailty index focuses on the cumulative impact of a patient’s clinical deficits identified by chronic diseases, signs, symptoms, and abnormal test results, allowing it to be quantified as a ratio (deficits present/total deficits considered) ranging from 0 to 1. In this context, the Rockwood frailty index (RFI) was calculated from 40 potential deficits (Collerton et al. [12]). Likewise, using the deficit accumulation index, several studies quantified frailty syndrome by means of shorter items’ scales such as the Carolina Frailty Index (CFI) based on 36 items [21], an 11-item modified Frailty Index (mFI), and the Frailty index containing 36 possible “health deficits”. Finally, a study applied the Edmonton Frailty Scale (EFS) to assess frailty [30]. This scale, an abbreviated assessment of the Comprehensive Geriatric Assessment, takes into account 10 domains (cognition, mood, functional independence, medication use, social support, nutrition, health attitudes, continence, burden of medical illness, and quality of life); its maximum score is 17 and represents the highest level of frailty [31].

The relationship between total lymphocyte counts and frailty percentages has also been evaluated in three longitudinal studies conducted in cancer patients [28,29] and institutionalized older women [32]. However, only one of them validated the associations observed in cross-sectional studies, showing that in addition to being associated with frailty at the beginning of the study, low lymphocyte counts predicted its progression at one year of follow-up, with a moderate sensitivity of 65.2% and a specificity of 68.7% [28].

A reduction in total lymphocyte count in blood is believed to be a characteristic marker of deleterious changes in the immune system associated with aging [33], and lymphocyte counts tended to decrease with age [34]. However, recent studies indicate that nearly every component of the immune system undergoes dramatic age-associated restructuring, leading to changes that include both enhanced as well as diminished function depending on the subtype of immune cells and their location [35]. Indeed, the emerging consensus is that immunological aging is part of a continuum of developmental processes, encompassing complex reorganizational events, compensatory mechanisms, and qualitative alterations in function. Confirming this, our analysis showed that only some of the subtypes of lymphocytes are associated directly or inversely associated with frailty syndrome.

**Table 1:** Main characteristics of clinical studies analyzed in frailty patients.

Reference Sorted by Year of Publication	Study Design	Sample Size (*n*)	Subjects (Sex and Age)	Participants	Frailty Assessment	Relationship between Total Lymphocyte Count and Frailty
Semba et al., 2005 [20]	Case-control study.	122	Community dwelling-women (cases) who died during 5 years of follow-up (mean age 76.9 ± 6,4 years) and women (controls) matched by age, frailty, and morbidities who survived during 7 years of follow-up (mean age 77.3 ± 6.8 years).	Community-dwelling adults.	Fried’s criteria.	There were no significant differences in counts or percentages of lymphocytes between frail, pre-frail, and non-frail women.
De Fanis et al., 2008 [23]	Case-control study.	26	22 women and 4 men with a mean age of 83.8 ± 5.3 years (range 72–94).	Community-dwelling adults.	Fried’s criteria	No significant differences in total lymphocyte counts between frail and non-frail participants were observed.
Leng et al., 2009 [10]	Observational cohort study.	1106	Women from the WHAS I cohorts with an age range of 65–102 years and women from the merged WHAS I and II cohorts with an age range of 70–79 years.	Community-dwelling woman.	Fried’s criteria.	No significant association between total counts of lymphocytes with frailty was identified.
Collerton et al., 2012 [12]	Cross-sectional study.	845	Different cohorts with a percentage of women in each cohort ranging from 60 to 75%. All participants were over 85 years old.	Community-dwelling or institutionalized older people.	Rockwood frailty index and Fried’s criteria.	The total lymphocyte count was inversely related to both measures of frailty, Fried scale and the Rockwood frailty index.
Fernández-Garrido et al., 2014 [16]	Cross-sectional study.	42	Women with an average age of 84.2 (±6.5) years (range, 70–99 years).	Non-demented institutionalized older population.	Fried’s criteria.	There was a significant and inverse relationship between the number of fulfilled frailty criteria and the lymphocyte count.
Nishijima et al., 2017 [21]	Cross-sectional study.	133	54 women and 79 men with a median age of 74 years (range 65–92).	Cancer patients.	36-item CFI.	Although the lymphocyte count in isolation was not related to frailty, the NLR was positively correlated with the frailty. Patients with a higher NLR also had increased odds of being frail/pre-frail.
Hou et al., 2018 [36]	Cross-sectional study.	345	154 women and 191 men with a median age of 71.0 years (IQR 65.0–77.0 years).	Elderly patients with coronary heart disease, (ACS (83.6%) and single-vessel disease (66.4%)).	Fried’s criteria.	A significant positive correlation was observed between NLR and the frailty score, and increased odds of being frail.
Fernández-Garrido et al., 2018 [32]	Two-year follow-up study.	94	Women with an average age of 82 (±7) years.	Non-demented institutionalized older women.	Fried’s criteria.	There was a significant inverse correlation between the frailty scores and lymphocyte counts at baseline, but not at follow-up.
Bernabeu-Wittel et al., 2019 [27]	Multicenter cohort study.	444	200 women and 244 men with an average age of 77.3 (±8.4) years.	Community-dwelling (93.7%) and institutionalized (6.3%) older patients (outpatients in the Internal Medicine and Geriatric areas).	Fried’s criteria.	The combined presence of frailty and sarcopenia was associated with a lower lymphocyte count.
Wilson et al., 2019 [19]	Observational cohort study.	377	185 women and 192 men with an average of 73.7 years (range, 50–98 years).	Patients hip fracture.	Modified 11-item frailty index score.	Total lymphocyte count weakly inversely correlated with frailty.
Navarro Martínez et al., 2019 [37]	Cross-sectional clinical trial.	46	Men with an average age of 72.2 (±9.4) years (range, 51–92 years).	Patients with prostate cancer undergoing antiandrogen therapy.	Fried’s criteria.	The lymphocyte counts were significantly lower in both frail and prefrail individuals than in robust individuals.
Marcos-Pérez et al., 2019 [38]	Cross-sectional study.	259	174 women and 85 men with an age range of 65–102 years.	Patients were contacted through associations of older or retired people, day care centers, and nursing homes.	Fried’s criteria.	The relationship between frailty and lymphocyte count was not studied in isolation.
Núñez et al., 2020 [18]	Observational study.	488	200 women and 188 men with an average age of 78 (±7) years.	Patients surviving an episode of an ACS.	Fried’s criteria.	The low percentage of lymphocytes was associated with frailty and a higher risk of being frail.
Buigues et al., 2020 [28]	One-year follow-up study.	39	Men with an average age of 71.9 (± 9.8) years.	Patients with prostate cancer undergoing antiandrogen therapy.	Fried’s criteria	At baseline lower lymphocytes count were significantly correlated with the frailty syndrome severity and predicted its progression at one year of follow-up.
Samson et al., 2020 [39]	Observational cohort study.	289	144 women and 145 men between 60–87 years of age.	Elderly people.	Frailty index containing 36 possible “health deficits”.	The relationship between frailty and the total lymphocyte count was not studied (they studied separately subpopulations of T cells, B cells, NK cells counts).
Bodolea et al., 2020 [26]	Observational cohort study.	179	101 women and 78 men with an average age of 65.07 (±12.9) years (range, 23–90 years).	Patients with cardiovascular disease	Fried’s criteria.	Lower lymphocyte count and NLR were significantly correlated with the frailty syndrome and its severity.
Bilgin et al., 2021 [30]	Observational cohort study.	108	57 women and 51 men. Median ages of the frail and non-frail groups were 65 (50–78) years and 62 (50–79) years, respectively.	Patients with type 2 diabetes mellitus.	Edmonton Frail Scale.	Elevated MPVLR were significantly correlated with the frailty syndrome and its severity.
Gilmore et al., 2021 [29]	Longitudinal cohort study.	581	Women. Age range, 22–81 years.	Women with stage I-IIIC breast cancer.	Fried’s criteria modified	Low lymphocyte counts and the NLR were associated with post-chemotherapy frailty, as well as changes in frailty from pre-chemotherapy to post-chemotherapy.

ACS: Acute coronary syndromes; CFI: Carolina Frailty Index; IQR: Interquartile range; MPVLR: Mean platelet volume/lymphocyte ratio; NLR: Neutrophil to lymphocyte ratio; WHAS: Women’s Health and Aging Study.

The inverse association between total lymphocyte counts in most of the analyzed studies and frailty syndrome can be attributed to several factors, including the decrease in immune surveillance and the replicative senescence that occurs during aging. In this context, elderly people with low lymphocyte counts may be more vulnerable to illness and early death and adverse outcomes associated with frailty. A low lymphocyte count may therefore not only indicate a depletion of the lymphoid cell line but could also be a marker for a decline in other physiological functions. If this is the case, a low lymphocyte count could reflect multisystem dysregulation associated with frailty.

Another reason that lymphocyte counts decline in frail people could be related to the release of glucocorticoids as a result of the activation of the hypothalamic-pituitary-adrenal axis in response to situations that threaten homeostasis, as occurs in chronic stress and persistent [3]. Several studies have shown that as well as a weak circadian variation, high cortisol levels may be involved in the development of frailty [40] and therefore contribute to a lower lymphocyte count in frail individuals.

The lower level of lymphocytes associated with frailty could also be mediated by the loss of appetite that occurs in frail people, who may not be aware of hunger due to the alteration of the hypothalamic feedback mechanism of leptin, or the release of proinflammatory cytokines [41]. A low nutritional intake, mainly proteins and some micronutrients, favors the deterioration of the immune response, and may induce a decline in the number of lymphocytes [34]. A significant volume of the literature in the area of malnutrition has used the total lymphocyte count as an indirect marker of nutritional status [42]. In contrast to this hypothesis, in a study carried out in a population of 377 patients with hip fractures, although frailty quantified by the modified frailty index was correlated with a lower total lymphocyte count, there was no association between frailty and a total lymphocyte count <1500 cells per cubic millimeter, used as a marker of malnutrition [19].

Low lymphocyte levels in frail people could also be related to the effect of physical exercise on the immune system, as reduced physical activity is one of the five criteria for frailty according to Fried [5]. To support this hypothesis, increased lymphocyte counts are positively associated with physical activity in young people [43], and lymphocyte production and physical activity performance appear to share common physiological pathways. Based on this hypothesis, Bernabeu Wittel et al. [27] observed in a hospital population of 444 polypathological patients that the combined presence of frailty and sarcopenia, defined as a loss of muscle mass and strength, was associated with both fewer lymphocytes and poor functional status.

In order to enhance knowledge of the cellular bases of the inflammatory state associated with frailty, four studies have explored the association between frailty and the neutrophil/lymphocyte ratio (NLR) [21,25,26,29], which is a more accurate marker of inflammation than the individual lymphocyte count, since it reflects the balance between two cellular markers of inflammation [36]. In a study carried out in cancer patients, the NLR was positively correlated with frailty, evaluated using the Carolina frailty index of 36 items [21]. In that study, participants in the upper tertile (>4.2) of the NLR also had almost 4 times the risk of being frail than people in the lower tertile (<2.5) of the NLR. Similarly, in a recent longitudinal study, Gilmore et al. [29] demonstrated that in women with breast cancer, RLN was associated with frailty after chemotherapy, as well as with the change in frailty from prechemotherapy to postchemotherapy, although this marker did not predict frailty 6 months after completing chemotherapy. Hou et al. [36] suggested that frailty was associated with this marker of inflammation in patients with coronary artery disease. In specific terms, they observed that an increase in NLR results in an increased risk of frailty. Another study found a higher NLR in frail patients with cardiovascular disease [26]. Although this marker of inflammation has not been studied in the general population of older adults, in a study carried out by Fernández-Garrido et al. [32] in institutionalized older women, the neutrophil and lymphocyte percentages were correlated with the frailty phenotype score in opposite directions. Similar results were observed in another cohort of people aged 85 or older living in the community, where frailty, measured using the Fried and Rockwood models, was associated with a decrease in lymphocyte count and a higher neutrophil count [12]. This shows that the mechanisms underlying the relationship of a high NLR with frailty could reflect the immunosuppressive effect of neutrophils on lymphocytes in chronic inflammatory responses, mediated by the release of pro-inflammatory cytokines produced by neutrophils. This hypothesis is corroborated by the large amount of evidence supporting the association between lymphopenia and inflammation [44]. The mean platelet volume and lymphocyte count, contributors to the mean platelet-volume-to-lymphocyte ratio (MPVLR), are also associated with inflammatory conditions. The role of this new inflammatory marker in frailty has also been studied by Bilgin et al. [30], who found that a higher MPVLR was associated with the condition of being frail and its severity in subjects with type 2 diabetes. Furthermore, an MPVLR value above 3.41% predicted frailty with high sensitivity (71%) and moderate specificity (51%).

### 3.2. Relationship between Lymphocyte Subtypes and the Presence of Frailty Syndrome and Its Severity

Five studies (Table 2) evaluated the relationship between the severity of frailty syndrome and the blood counts of lymphocyte subpopulations in community-dwelling older adults. They found an association between frailty and increased numbers of CD8+ lymphocytes [12,17,20] and in the CD8+CD28-lymphocyte ratio and with a lower count of lymphocyte CD4+/CD8+ ratio [20], which represents a marker of aging of the immune system [39,45]. Interestingly, both frailty and citomegalovirus seropositivity were associated with an increase in CD28-T cell counts. An epidemiological study suggested that CMV seropositivity is an independent risk factor for frailty in older women [46]. The increased ratio between memory and naïve CD8 T cells was replicated using both Fried’s and Rockwood’s frailty scale in community-dwelling older adults in the Newcastle 85+ study [12]. In contrast, the memory/naïve CD4 T cell ratio, the CD4/CD8 T cell ratio, and the memory/naïve B cell ratio were not significantly associated with frailty syndrome in this study when their blood counts were compared with the severity of frailty measured by Fried’s scale, and the results partially overlapped when Rockwood’s frailty index was also taken into account [12]. The association between frailty syndrome and B cell counts was only reported in two studies, with the Rockwood frailty scale in one study [12] and Fried’s scale in another one [17]. In a subcohort study performed in older adults belonging to the Doetinchem cohort study in the Netherlands, the frailty syndrome was associated with some lymphocyte subpopulations among the 37 subpopulations studied [30]. Frailer women, but not men, showed fewer T lymphocytes, and CD56+ T cells in particular [30], which are known for their cytotoxic capacity [22]. This result is consistent with another study reporting an increased expression of CD56 in lymphocyte T cells from individuals with better cognitive and physical functioning [47]. Frailty syndrome was also associated with significantly fewer late differentiated CD4+ TemRA T cells in older frail women and represent a subset of effector memory T cells expressing CD45RA (termed TEMRA) after antigenic stimulation, which display a transcriptional and proteomic program with cytotoxic features [47]. Cells in the immune system that express receptors for chemokines participate in the regulation and effectors of the immune system. The expression of different chemokine receptors has been associated with Th1 and Th2 lymphocyte phenotypes, with Th1 cells expressing CXCR3 and CCR5, while Th2 cells express CCR3 and CCR4 [48]. Frailty syndrome has also been associated with an increase in some subpopulations of T lymphocytes expressing a subtype of CCR receptors. In fact, higher concentrations of total CCR5, CCR5-CD8, and CCR5-CD45RO T cells (called “memory” lymphocytes) have been described in frail older adults than in non-frail controls [26], suggesting a significant expansion of a specific subset of T cells with a proinflammatory type 1 phenotype [49]. CC chemokine receptor 5 (CCR5) interacts with CCL3, 4, 5, and 8 chemokines, and plays an important role in the regulation of leukocyte recruitment, trafficking, and immune activation [50]. CCR51 T lymphocytes have been shown to have a proinflammatory type 1 phenotype, and CCR5+ T lymphocytes can significantly contribute to several inflammatory conditions such as frailty syndrome and related adverse outcomes [23,51].

Although the identification of lymphocyte counts as a possible biomarker for a single frailty criterion may be very useful for understanding the specific mechanisms responsible for the development of frailty, few studies have evaluated the relationship between lymphocyte counts with each of the five frailty criteria on the Frail scale [13,21,24,26]. Likewise, lymphocyte percentages have been found to be significantly related to low levels of physical activity and lower muscle strength in a population of institutionalized older women [16]. These two measures directly or indirectly reflect a decline in muscle activity. The main component of age-related muscle deterioration is sarcopenia, which is defined as a progressive and generalized loss of skeletal muscle mass and strength, and this condition is in turn considered a key component in the pathophysiology of frailty syndrome in older adults [37]. Various studies suggest that sarcopenia is greater in frail older people than in non-frail older people [38]. Given that sarcopenia, like frailty syndrome, involves the dysfunction of different interrelated physiological systems, it is conceivable that the mechanisms leading to sarcopenia often overlap with those of frailty syndrome [52]. Chronic systemic inflammation has been implicated in the development of both frailty and sarcopenia, and several inflammatory mediators have been suggested as contributors to both sarcopenia and frailty [27,53,54]. Likewise, in terms of cellular markers of inflammation, a study carried out in a population of hospitalized polypathological patients observed that lower lymphocyte counts were factors independently associated with the presence of both conditions [27]. The mechanism by which low lymphocyte counts are associated with a low level of physical activity may be related to the effects of physical exercise on the immune system. A higher level of physical activity is associated with an increase in the lymphocyte count in healthy older adults [55]. The underlying mechanisms are multifactorial and include neuroendocrine and metabolic factors [56]. However, this relationship between exercise and lymphocyte counts has not been demonstrated in frail older adults [57]. It has been suggested that this may be due to the fact that older adults, once they become frail, may not be able to perform sufficient levels of exercise to cause changes in immunity, or that exercise cannot reverse immune changes associated with this state [58]. However, it is difficult to draw conclusions since the available data are too limited. The mechanism by which low lymphocyte counts are associated with lower levels of physical activity in frail older adults has therefore not been identified. Further studies, including functional studies of specific lymphocyte subtypes, are needed to assess the role of lymphocytes more fully in physical activity and vice versa in frail older adults.

On the other hand, the effect of low lymphocyte levels on lower muscle strength can be attributed to the immunosuppressive effect of pro-inflammatory cytokines caused by neutrophils on cytotoxic T lymphocytes [44]. In a recent study of a population of older adults with coronary heart disease, reduced muscle strength and slow gait speed were associated with an increase in NLR, defined as the absolute count of neutrophils divided by the count of lymphocytes [26]. Furthermore, in elderly cancer patients, a higher NLR was significantly associated with lower scores for instrumental activities of daily living and in the prolonged Timed Up and Go Test (≥14) [21]. This nonspecific cellular marker of systemic inflammation could reflect the relationship in opposite directions between reduced muscle strength and lymphocyte and neutrophil counts observed in a cross-sectional study of older people [32]. In addition to favoring a lower production of lymphocytes, by inducing a chronic inflammatory response, the production of free radicals and pro-inflammatory cytokines by neutrophils can cause damage to multiple tissues, including muscle tissue [58,59]. The activation of pro-inflammatory cytokines promotes anorexia and protein catabolism of skeletal muscle, which could contribute to a worsening of the nutritional status and a reduction in muscle mass and consequently to muscle weakness cause, leading in turn to a slow gait speed and less physical activity [1]. Furthermore, this state of chronic inflammation leads to the appearance of endothelial dysfunction, insulin resistance, and procoagulant phenomena, all of which are promoters of arteriosclerosis [60]. Arteriosclerosis leads to an alteration in the perfusion and therefore to a decline in the irrigation of the skeletal muscles, which reduces the availability of oxygen in them and aggravates sarcopenia, and consequently leads to a loss of muscle strength.

Finally, significant differences between self-reported fatigue and lymphocyte counts were also observed in a population of older men with prostate cancer undergoing androgen deprivation therapy, with subjects reporting low energy presenting lower lymphocyte counts [13]. It has been suggested that the pathways that lead to fatigue after cancer are related to the disease itself, to other diseases, to treatment, or to the psychological reaction to the disease. Anxiety and depression are the most common psychological causes of fatigue in cancer patients. The evidence also shows a functional relationship between stress and immunity mediated by neuroendocrine factors, such as the release of glucocorticoids as a result of activation of the hypothalamic-pituitary-adrenal axis [61]. There is a close relationship between the leukocyte concentration curve and the level of plasma glucocorticoids during physiological stress, since when they act, these hormones increase the number and percentage of neutrophils, while lymphocytes are reduced [62,63]. Likewise, depression has highlighted the existence of a biochemical profile at the immunological level similar to that observed in stress [64]. In a population of older women, a trend toward a reduced lymphocyte count was observed in depressed patients compared to non-depressed patients [65]. Based on this, it is easy to understand that mood disturbances can contribute to a state of chronic systemic inflammation and ultimately to the development of self-reported fatigue seen in frail people with cancer.

## 4. Conclusions

A lower total count or percentage of lymphocytes has been associated with frailty and its severity in different populations of older adults. These findings provide additional support for theories linking inflammation to frailty and provide insight into the potential roles of lymphocytes in the pathogenesis of frailty. Likewise, the inclusion of this common analytical parameter in traditional frailty indices could help to identify frailty, even at an early stage, and its monitoring would enable assessment of the progression of frailty and the response to the applied interventions.

## Figures and Tables

**Table 2:** Lymphocyte subtypes and their relationship with frailty.

Reference Sorted by Year of Publication	Study Design	Sample Size (*n*)	Subjects (Sex and Age)	Disease/Patients	Frailty Definition	Lymphocyte Subtypes Studied	Relationship between Lymphocyte and Subtypes Count and Frailty
Semba et al., 2005 [20]	Case-control study	61 women who died (cases) to 61 women who did not die (controls) during follow-up were matched	Women (cases) with a mean age of 76.9 (6.4) years and women (controls) 77.3 (±6.8) years.	Community-dwelling adults.	Fried’s criteria.	Counts or percentages of CD4+, CD8+, CD4+CD28-, CD4+CD28+, CD8+CD28-, CD8+CD28-, CD4+CD45RA+, CD4+CD45RO+, CD8+CD45RA+, CD8+CD45RO+ T cells and CD4/CD8 T cells ratio.	Frail women appeared to have significantly higher CD8+ and CD8+CD28− lymphocyte counts. Frail women also had significantly lower CD4+, lower CD4+CD28+, higher CD8+, higher CD8+CD28-, and lower CD8+CD28+ percentages
De Fanis et al., 2008 [23]	Case-control study	26 frail and no frail participants were matched.	84,6 % were women and 15.4% men with a mean age of 83.8 ± 5.3 years (range 72–94).	Community-dwelling adults.	Fried’s criteria	Counts of CD3+, CD4+, CD8+, CD45RO+,CD45RO-, CCR5+, CCR5+ CD4+, CCR5+CD8+, CCR5+CD45RO+ and CCR5+CD45RO-T cells.	Frail participants had higher CCR5+, CCR5+CD8+, and CCR5+CD45RO- T-cell counts than matched non-frail controls.
Collerton et al., 2012 [12]	Cross-sectional study	845 patients.	+85 year old.	Community-dwelling or institutionalized older people.	Rockwood frailty index and scale Fried.	Count and ratios of CD4/CD8 T cells, memory/naïve CD4 and CD8 T cells and memory/naïve B cells.	High levels lymphocytes memory/naïve CD8 T cell ratio were associated with a lower risk of frailty on the Fried scale and low levels of memory/naïve B cells were associated with a higher risk of frailty on the Rockwood frailty index.
Marcos-Pérez et al., 2019 [38]	Cross-sectional study	259 patients.	85 male and 174 female with an age range of 65–102 years.	Patients were contacted through associations of older or retired people, day-care centers, and nursing homes.	Fried’s criteria.	Percentages of CD3+, CD4+ and CD8+ T cells, CD19+ B cells, CD16+56+ NK cells and CD4/CD8 T cells ratio.	A significant increase in the CD4+/CD8+ ratio and a significant decrease in the % CD19+ cells were observed in the frail group.
Samson et al., 2020 [39]	Observational cohort study.	289 patients.	145 men and 144 women between 60–87 years of age.	Elderly people.	Frailty index with incorporates 36 possible “health deficits”	The numbers of CD16 and CD56 NK cells, CD56+ T cells and CCR7 + CD4 +/CD8 T cells, which were classified as naïve (CCR7+CD45RA+) or central memory (CCR7 + CD45RA-) T cells. CCR7-CD4 +/CD8 + T cells were divided into effector memory T cells (Tem, CCR7-CD45RA-) and effector memory T cells that re-express CD45RA T cells (TemRA, CCR7-CD45RA +).	More frail women, but not men, showed fewer CD56 + T cells and fewer CD4 + TemRA cells.

## Data Availability

Not applicable.
